# Mutation Analysis of* HTRA2* Gene in Chinese Familial Essential Tremor and Familial Parkinson's Disease

**DOI:** 10.1155/2017/3217474

**Published:** 2017-01-24

**Authors:** Ya-Chao He, Pei Huang, Qiong-Qiong Li, Qian Sun, Dun-Hui Li, Tian Wang, Jun-Yi Shen, Juan-Juan Du, Shi-Shuang Cui, Chao Gao, Rao Fu, Sheng-Di Chen

**Affiliations:** Department of Neurology and Collaborative Innovation Center for Brain Science, Ruijin Hospital, Shanghai Jiao Tong University School of Medicine, Shanghai 200025, China

## Abstract

*Background*.* HTRA2* has already been nominated as PARK13 which may cause Parkinson's disease, though there are still discrepancies among these results. Recently, Gulsuner et al.'s study found that* HTRA2* p.G399S is responsible for hereditary essential tremor and homozygotes of this allele develop Parkinson's disease by examining a six-generation family segregating essential tremor and essential tremor coexisting with Parkinson's disease. We performed this study to validate the condition of* HTRA2* gene in Chinese familial essential tremor and familial Parkinson's disease patients, especially essential tremor.* Methods*. We directly sequenced all eight exons, exon-intron boundaries, and part of the introns in 101 familial essential tremor patients, 105 familial Parkinson's disease patients, and 100 healthy controls.* Results*. No exonic variant was identified, while one exon-intron boundary variant (rs2241028) and one intron variant (rs2241027) were detected, both with no clinical significance and uncertain function. There was no difference in allele, genotype, and haplotype between groups.* Conclusions*.* HTRA2* exonic variant might be rare among Chinese Parkinson's disease and essential tremor patients with family history, and* HTRA2* may not be the cause of familial Parkinson's disease and essential tremor in China.

## 1. Introduction

As two of the most prevalent tremor disorders, essential tremor (ET) and Parkinson's disease (PD), which are estimated to constitute 0.9% and 0.3% of worldwide population, respectively, are considered as distinctively different entities formerly [1, 2]. Several lines of evidence showed that there are remarkable overlaps in clinical features, epidemiology, imaging, genetics, and pathology between PD and ET, including a fourfold increase of risk developing Parkinson's disease in essential tremor cases [3, 4].

ET is widely regarded as caused by genetic with no disease-causing gene ever been focused; Contrarily, though PD is mainly sporadic, up to now 22 PARK loci have been identified [5, 6]. To be specific, 50% of ET patients demonstrate familial aggregation, while less than 15% of PD patients have affected first-degree relatives [7–9]. Due to the overlap phenomena between ET and PD, investigations into the relationship between PD risk variants and ET patients have been done, involving* LINGO1, LINGO2, LRRK2, SLC1A2,* and* HTRA2* genes [3, 10–12].

HTRA2 has already been nominated as PARK13 which may cause Parkinson's disease, though there are still discrepancies among these results. Recently, a research by Gulsuner and colleagues examining a six-generation family segregating ET and ET coexisting with PD revealed that* HTRA2* p.G399S is responsible for hereditary essential tremor and homozygotes for this allele develop Parkinson's disease [13]. Replications conducted in Western Norway and Asian population to address the association between p.G399S and ET, PD, ET/PD, and tremulous cervical dystonia failed to reach a consensus [14, 15]. In addition, report from a small sample (29 FETs) in Germany adopting coding exon Sanger sequencing did not reconfirm it either [16]. To validate the condition in Chinese familial essential tremor (FET) and familial Parkinson's disease (FPD) patients, we performed a Sanger sequencing of eight exons and exon-intron boundaries of* HTRA2* instead of just one variant (p.G399S).

## 2. Methods

### 2.1. Patients

This study enrolled* 221* unrelated Chinese patients, including* 105 PD* patients with autosomal dominant inheritance (2 or more affected relatives in 2 consecutive generations),* 101 ET* patients with family history, and* 15* patients of ET coexisting with PD. All patients were from the Movement Disorder Clinic of Department of Neurology at Ruijin Hospital affiliated to Shanghai Jiao Tong University School of Medicine. PD and ET patients were diagnosed by senior movement disorder specialist on the basis of MDS clinical diagnostic criteria for Parkinson's disease and Consensus Statement on Tremor of the Movement Disorders Society, respectively [17, 18]. Patients presenting secondary Parkinsonism, Parkinson-plus syndrome, or hyperthyroidism were excluded from the study. We also included* 100* healthy controls without any symptom of movement disorders. The demographic information of patients is shown in Table 1. We received approval from the Ethics Committee of Ruijin Hospital affiliated to Shanghai Jiao Tong University School of Medicine. Written informed consents were obtained from all patients and controls participating in the study as well.

### 2.2. DNA Sequencing and Mutation Analysis

Genomic DNA was extracted from venous blood applying standardized phenol/chloroform extraction method from patients and controls. The 8 coding sequences, exon-intron boundaries, and part of introns were sequenced by Sanger sequencing in 4 products of PCR (polymerase chain reaction) amplification using 4 pairs of primers (Table 2). DNASTAR Lasergene MegAlign (v7.1.0) and Chromas (v2.33) were used to conduct sequence alignment, and the chromatograms were double checked to avoid missing any variants. Variants detected were searched in NCBI to get access to their clinical significance and MAF in ExAC and 1000 Genomes Projects database.

### 2.3. Statistical Analysis

Statistical analysis was performed with Statistical Analysis System V8 (SAS V8). Difference of age was assessed applying *t*-test or *t*′-test. Hardy-Weinberg equilibrium (HWE) was calculated by Chi-square analysis. Chi-square or Fisher's exact test was used to test the differences in genotype and gender between groups. Odds ratios (ORs) and 95% confidence intervals (95% CI) were evaluated by Mantel-Haenszel Chi-squared test to verify the association between variants and FPD or FET. The evaluation of the association was also conducted using logistic regression under different genetic models adjusted for age and gender. Online SHEsis program was used to conduct haplotype analysis [19]. Two-tailed *p* value < 0.05 was considered significant. The statistical power was performed using Quanto.

## 3. Results

The patients and controls in the study are well matched for mean age (*p* = 0.12 for FET and *p* = 0.86 for FPD) and sex distribution (*p* = 0.52 for FET and *p* = 0.52 for FPD) (Table 1). By sequencing all the four products in all 221 patients (FET, FPD, and ET-PD) and 100 controls, no exonic variant was identified, while one exon-intron boundary variant (rs2241028) and one intron variant (rs2241027) were detected. In NCBI SNP database, MAF of rs2241027 and rs2241028 were 0.05/0.10, 0.06/0.07, respectively, from ExAC/1000 Genomes Project, both with no clinical significance. The function of both variants was defined as uncertain by MyGenostics. The variants distribution was within the range of Hardy-Weinberg equilibrium in controls (*p* = 0.82, 0.71 resp., Table 3). Given the present sample sizes, we have 80% power to detect an odds ratio of 1.83 in both PD and ET for rs2241027 adopting an additive model and OR of 1.91 in both PD and ET for rs2241028 adopting an additive model. What is worth noting is that there are big differences in MAFs between our control and database in both two variants, which may be caused by ethnical diversity, so we calculate the power considering MAFs of 0.28 and 0.21, respectively, in our control, which is higher than in database; otherwise, it would require much bigger sample sizes. Additionally, we only have 34% power to detect an OR of 1.44 (the OR in Krüger et al.'s study) for rs2241028.

As for allele and genotype distribution of both variants, we failed to detect any significant differences either in FET versus controls or in FPD versus controls (Tables 3 and 4). No significant difference was observed in the logistic regression either (data not shown). Moreover, haplotypes of two variants showed hardly any association with the risk of FET or FPD (Table 5). Regarding ET-PD, in which we attempted to investigate the situation of* HTRA2* in case there were some dramatic mutations, owing to the limitation of sample size, we quitted further statistical analysis.

## 4. Discussion

The high temperature requirement A2 (*HTRA2*), known as a mitochondria protein, plays distinct different roles in mitochondria homeostasis and cellular apoptosis regulation [20]. As one study indicated, deficiency of* HTRA2* can cause damage and mutation of mitochondria DNA [21]. Another study revealed that* HTRA2* was regulated by* PINK1*, which might contribute to early-onset PD, in the proteolytic activity [22].

Many researches concerning the association of PD with* HTRA2* variants have been done. The earliest mutation screening of* HTRA2* in PD patients was done in a German population after the finding that targeted disruption of HTRA2 can cause neurodegeneration and a Parkinsonian phenotype in mice, which resulted in the identification of two mutations (G399S and A141S) related to the risk of PD [23, 24]. Later on, replications with contradictory consequences have been conducted [25–31], and one large scale genetic association study is worth noting, which showed no evidence for an overall association of common variants in* HTRA2 *with PD [32], while Gulsuner et al.'s study of a six-generation family provides further evidence for the probability of* HTRA2* acting as a cause for PD and ET, especially those with family history [13]. So the aim of our study is to investigate the situation of* HTRA2* by Sanger sequencing of the whole coding sequence in FET, FPD, and ET-PD in Chinese population, especially FET and ET-PD.

Our study detected two variants (rs2241028, rs2241027). Variant rs2241028 has been reported in several studies with similar negative results except for Krüger et al.'s study, in which rs2241028 was considered as a susceptible factor for PD in the Scandinavian population and their descent from USA [32], while there is no report of this variant in studies about ET. Since rs2241028 is near the splicing region, it may affect the transcript efficiency of HTRA2 to some extent or influence the expression of HTRA2 in some other way, so it would be promising to do some research into the function of this variant and the relationship with PD. Variant rs2241027 has never been mentioned in the previous study no matter about PD or ET. Our study showed that neither of two variants was related to the risk of developing ET or PD, and two variants were defined as no clinical significance in database. Meanwhile, we have not detected mutations (G399S and A141S) mentioned in other studies. So we provided no evidence of association of* HTRA2* with FET and FPD. As for ET-PD, the result of our study was not so convincing due to the sample size though we found nothing significant as well. Admittedly, there are some limitations in our study. On the one hand, the sample sizes were only able to detect a moderate correlation with enough power and not for a relatively weaker correlation, which may cause false negative error. On the other hand, it would be more persuasive if the promoter of HTRA2 gene has been sequenced as well.

In conclusion,* HTRA2* might not be a cause of familial ET or PD in China. Studies with larger sample size are needed to investigate thoroughly the role of* HTRA2* in ET and ET-PD in China and other places in the world.

## Figures and Tables

**Table 1:** Demographics of participants.

Details	FET	FPD	ET-PD	Control
Total	101	105	15	100
Age^a^ (range, *p*^b^ value)	61.24 ± 12.62 (28–90, 0.12)	59.28 ± 11.21 (36–84, 0.86)	67.80 ± 8.65 (56–79, NA)	59.06 ± 6.21 (49–74, NA)
Male/female, *p*^b^ value	51/50, 0.52	53/52, 0.52	12/3, N/A	46/54, N/A

N/A: not applicable; ^a^data are mean ± SD; ^b^data are compared with control; FPD: familial PD; FET: familial ET; ET-PD: ET coexisting with PD.

**Table 2:** Primers of HTRA2.

Name	Forward	Reverse	Products
1	GTC TCA CAA CTC GCG TCC G	GCC TGA AAT GGA GGG AAA GCA	Exon 1 and boundaries
2	TCG AGA TCC TGG ACC GGT AA	GGC CAC ATT TTT GCA GCC TAA	Exons 2, 3 and intron 2; boundaries
3	GCA GCT ATT GAT GTG CGT CC	TGA AGG GAG ACA GCT CTT GTG	Exons 4, 5, 6 and introns 4, 5; boundaries
4	ACT CAG CCA ACC TGA TTT CCT AC	TTC AGA GCC CAG GAG TCA GT	Exons 7, 8 and intron 7; boundaries

**Table 3:** Statistics of minor allele frequency (MAF).

RS number	Position	Function	MAF				ExAC/1000	HWE (*p*^a^)	OR (95% CI), *p* value	
			FET	FPD	ET-PD	Control	Genomes MAF		FET versus control	FPD versus control
rs2241027	Intron	Uncertain	0.33	0.29	0.33	0.28	0.05/0.10	0.82	1.28 (0.83–1.96), 0.26	0.80 (0.47–1.35), 0.40
rs2241028	Exon-intron boundary	Uncertain	0.14	0.16	0.23	0.21	0.06/0.07	0.71	1.08 (0.70–1.66), 0.73	0.70 (0.42–1.16), 0.17

^a^HWE for controls.

**Table 4:** Statistics of genotype.

Participants	Genotype (rs2241027/rs2241028)			*p* ^a^ value	
	GG	GA	AA	rs2241027	rs2241028
FET	44/75	48/23	9/3	0.50	0.29
FPD	53/73	43/31	9/1	0.77	0.14
ET-PD	8/9	4/5	3/1	N/A	N/A
Control	51/64	43/30	6/6	N/A	N/A

^a^ *p* value compared with controls.

**Table 5:** Haplotype analysis.

Haplotype	FET (%)	FPD (%)	Control (%)	*χ* ^2^ value^a/b^	Fisher's *p*^a/b^	Pearson's *p*^a/b^	OR (95% CI)^a/b^
A-A	0	0	0	—	—	—	—
A-G	33	29	28	1.28/0.12	0.26/0.73	0.26/0.73	1.28 (0.83–1.96)/1.08 (0.70–1.66)
G-A	14	16	21	3.05/1.92	0.08/0.17	0.08/0.17	0.63 (0.38–1.06)/0.70 (0.42–1.16)
G-G	53	55	52	0.09/0.58	0.77/0.45	0.77/0.45	1.06 (0.72–1.57)/1.16 (0.79–1.71)

^a/b^value for FET versus controls/FPD versus controls.
